# Microtechnologies for studying the role of mechanics in axon growth and guidance

**DOI:** 10.3389/fncel.2015.00282

**Published:** 2015-07-27

**Authors:** Devrim Kilinc, Agata Blasiak, Gil U. Lee

**Affiliations:** Bionanosciences Group, School of Chemisty and Chemical Biology, University College DublinBelfield, Dublin, Ireland

**Keywords:** growth cones, cytoskeleton, mechanotransduction, microenvironment, axon towing

## Abstract

The guidance of axons to their proper targets is not only a crucial event in neurodevelopment, but also a potential therapeutic target for neural repair. Axon guidance is mediated by various chemo- and haptotactic cues, as well as the mechanical interactions between the cytoskeleton and the extracellular matrix (ECM). Axonal growth cones, dynamic ends of growing axons, convert external stimuli to biochemical signals, which, in turn, are translated into behavior, *e.g*., turning or retraction, *via* cytoskeleton–matrix linkages. Despite the inherent mechanical nature of the problem, the role of mechanics in axon guidance is poorly understood. Recent years has witnessed the application of a range of microtechnologies in neurobiology, from microfluidic circuits to single molecule force spectroscopy. In this mini-review, we describe microtechnologies geared towards dissecting the mechanical aspects of axon guidance, divided into three categories: controlling the growth cone microenvironment, stimulating growth cones with externally applied forces, and measuring forces exerted by the growth cones. A particular emphasis is given to those studies that combine multiple techniques, as dictated by the complexity of the problem.

## Introduction

The guidance of axons to their proper targets is not only a fundamental event in the development of the nervous system, but also a potential therapeutic target for repairing the injured nervous system. A better understanding of axon guidance mechanisms may lead to novel therapies for neurological diseases, such as spinal cord injury or peripheral neuropathy. Axon guidance during development is orchestrated by numerous chemo- and haptotactic cues, and the timely expression of their appropriate receptors. Growth cones, the dynamic ends of growing axons, perceive attractive and repulsive cues and translate them into behavior, such as elongation, retraction, or turning (Kalil and Dent, 2005). The complexity of this chemotropic response becomes exhausting considering that growth cones also respond to various growth factors, developmental morphogens, neuromodulators, and extracellular matrix (ECM) proteins. Despite being tightly regulated by biochemical signals, axonal growth is essentially a series of mechanical processes that eventually drive the growth cone forward or cause its collapse. These processes involve the continuous rearrangement of cytoskeleton, actin filaments (F-actin) and microtubules, in particular (Lowery and Van Vactor, 2009). Force is generated through directed polymerization of cytoskeletal filaments, a process regulated by a range of associated proteins (Dent and Gertler, 2003), or by molecular motors operating on multiple filaments (Geraldo and Gordon-Weeks, 2009). Internally generated forces may simply push the cell membrane forward or pull on transmembrane adhesion molecules, which form focal adhesion complexes and link the cytoskeleton to the ECM or to other cells. These mechanical linkages are believed to be where mechanotransduction, *i.e*., the conversion of mechanical stimuli into biochemical signals, and* vice versa*, takes place.

Although the important role mechanics plays in axon guidance is more and more widely accepted, a thorough understanding of underlying mechanisms is lacking (Franze et al., 2013). We argue that this is largely due to the low accessibility of the experimental methods that permit mechanical probing of growth cones. Progress is being made in three distinct, yet overlapping aspects of the problem: controlling the growth cone microenvironment; applying forces to growth cones; and measuring forces generated in and/or exerted by growth cones. Since growth cones actively generate forces and are highly sensitive to the mechanical and other properties of their substrates, an ideal experimental model would integrate all three aspects of the problem in conjunction with biochemical stimulation. This way, mechanochemical inputs can be directly linked with mechanochemical outputs, and a comprehensive map of interactions can be laid out. In this mini-review we discuss novel microtechnologies that permit such integrative approach: engineered culture systems with controlled stiffness, topography, confinement, or protein patterning, and biophysical tools for applying or quantifying forces. We conclude by highlighting recent technologies that are yet to be translated into the axon guidance field.

## Controlling the Growth Cone Microenvironment

The complexity of axon growth and guidance phenomena led to the development of minimalistic *in vitro* models, where the neuronal microenvironment is tightly controlled. The substrate stiffness is typically controlled by adjusting the cross-linker concentration in a polymer, *e.g*., polyacrylamide, whose surface can be bio-functionalized to promote adhesion. Central (CNS) and peripheral (PNS) nervous system neurons exhibited different behavior on softer polyacrylamide compared to harder: PNS axons elongated less (Koch et al., 2012), whereas CNS axons formed more branches (Flanagan et al., 2002). The growth behavior also depended on the polymer material and whether neurons were cultured on 2D layers or within 3D matrices (Balgude et al., 2001; Blewitt and Willits, 2007). 3D collagen gels with varying stiffness can be created by imposing a crosslinker concentration gradient. When cultured in this gel, PNS axons grew longer down the gradient, *i.e*., towards softer regions (Sundararaghavan et al., 2009). Examples of biomimetic 3D matrices include silk nanofiber (Dinis et al., 2014) and salmon fibrin (Ju et al., 2007) gels. Despite the tremendous effort put into developing 3D porous hydrogels as conduits for spinal cord repair, the effects of gel mechanical properties on axon growth remain largely unknown. This is due to the difficulty of isolating stiffness from other parameters, such as porosity and degradation rate (Macaya and Spector, 2012). A promising new material is the self-assembling peptide nanofiber gels, which promote neural growth when embellished with laminin-like sequences (Sur et al., 2012). The stiffness of these gels can be tuned by simply changing the sequence of the β-sheet-forming peptide, *i.e*., independent of polymer density or cross-linker ratio (Sur et al., 2013).

**Figure 1:**
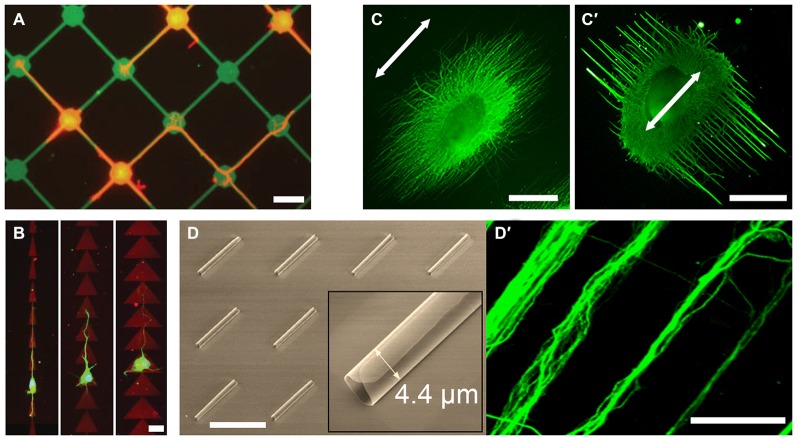
**Examples of novel growth cone microenvironments. (A)** Rat hippocampal neurons (stained for tubulin; red) adhere preferentially to 15 μm nodes and 2 μm wide stripes of fluorescein isothiocyanate-conjugated poly-l-lysine (FITC-PLL; green), pattered *via* direct photolithography and lift-off method. Adapted with permission from Withers et al. (2006). **(B)** Rat hippocampal neurons (stained for tubulin; green) grown on “fast-forward” patterns of polylysine (red) on a polyethyleneglycol monolayer, prepared *via* laser micropatterning. Adapted from Scott et al. (2012) with permission from The Royal Society of Chemistry. **(C,C′)** Chick dorsal root ganglia (DRG; stained for neurofilaments; green) cultured on electrospun poly-(ε-caprolactone) nanofiber gels with different fiber collection times and support substrates, but with the same fiber alignment (arrow). Adapted with permission from Xie et al. (2014). **(D)** Scanning electron micrograph of the self-rolled-up silicon nitride microtubes array and **(D′)** aligned bundles of mouse cortical axons (stained for tubulin; green). Adapted with permission from Froeter et al. (2014). Copyright 2014 American Chemical Society. Scale bars = 20 μm **(A,B)**, 1 mm **(C)**, 50 μm **(D)**.

While stiffness has an immediate effect on the growth cone–substrate mechanical coupling, geometrically restraining focal adhesions offers alternative means of control. Substrates decorated with patterns of adhesive regions (Hardelauf et al., 2014) or with microtopographical features (Hoffman-Kim et al., 2010) modulate neuronal polarity and guide axons. Patterns of adhesive regions can be created *via* microcontact printing, where molecules of interest are transferred to the substrate using an elastomeric stamp (Vogt et al., 2004). Alternative approaches include reactive plasma etching, where the stamp is used to protect a pattern from being etched away due to oxygen plasma (Kim et al., 2012), electron-beam lithography using adhesive and non-adhesive self-assembled monolayers (Yamamoto et al., 2012), and the “lift-off” method, where a patterned sacrificial layer is used to remove parts of coating. The latter was used to generate intricate patterns of polylysine (Figure 1A) to the study cytoskeletal organization in axon collateral branching (Withers et al., 2006). Multi-step patterning can be used to create complex patterns of multiple cell adhesion molecules (CAMs), which selectively promote axonal or dendritic growth (Shi et al., 2007). Alternatively, microdroplet printing can be used to pattern multiple ECM proteins to study the cooperation between ligands of different affinity (Féréol et al., 2011). Submicron feature sizes can be achieved *via* photopatterning, crosslinking of functional groups on a non-adhesive monolayer using a focused femtosecond laser. On photopatterned triangular isles of polylysine, axons preferentially grew in the “fast-forward” direction (Figure 1B; Scott et al., 2012). Finally, micropatterning is not limited to adhesive molecules: axonal guidance cues (von Philipsborn et al., 2006) and membrane-permeant analogs of second messengers that are downstream of these cues (Shelly et al., 2010) have been successfully patterned.

2D microtopography is typically created *via* photolithography, where large features, *e.g*., corridors and chambers, act as geometric constraints that guide axons (Francisco et al., 2007), whereas small features may act as structural handles. Anisotropic features, *e.g*., linear or circular grooves, promote unidirectional growth; whereas isotropic features, *e.g*., uniform micropillar arrays, result in higher elongation rates (Li et al., 2015). Forming contact with a structural handle is sufficient for an early stage neuron to polarize, as evidenced by N-cadherin clustering at the contact site, where an axon is subsequently initiated (Micholt et al., 2013). Contact-mediated signaling was also observed at isolated anchorages, *e.g*., carbon nanotube isles, where increased axonal tension resulted in synaptophysin-rich axon terminals. (Anava et al., 2009). Topography is suggested to modulate growth cone-substrate mechanical interactions at the molecular level (Moore and Sheetz, 2011). Accordingly, interaction with nanoscale anchorages increased focal adhesion density (thus traction force) in growth cone filopodia, suggesting a curvature-sensing mechanism based on asymmetric torque generation across the growth cone (Spedden et al., 2014). In 3D, nanotopography is readily introduced in electrospun nanofiber gels. Offering a variety of polymer formulations and biofunctionalization, these uniaxial scaffolds promote axon alignment and elongation (Schnell et al., 2007; Jin et al., 2011; Dinis et al., 2014). Interestingly, adjusting gel parameters, such as nanofiber density, induced axon alignment perpendicular (and not parallel) to the nanofiber orientation (Figures 1C,C′; Xie et al., 2014). Perpendicular (ladder) but not parallel (rope) growth was myosin-2-dependent, suggesting that nanoscale features are more than structural handles for growth cones.

Microfluidic devices provide additional means of microenvironmental control, such as imposing chemical gradients or fluidically isolating subcellular structures. These are important aspects of axon guidance *in vivo*, where growth cones experience different chemical environments than their somata and are typically exposed to chemotactic gradients. Gradients of substrate-bound cues can be imposed using flow-based devices (Dertinger et al., 2002); whereas gradients of freely-diffusing cues can be imposed using flow-based (Taylor et al., 2015) or diffusion-based (Dupin et al., 2015) devices. The latter can be achieved by flanking a culture chamber with source and sink channels and connecting them with narrow microchannels. Importantly, if the microchannels are narrow enough to permit the crossing of axons but not somata, axons can be fluidically isolated (Taylor et al., 2005). Bicompartmental devices thus allow probing growth cone-substrate interactions independent of other factors (Hur et al., 2011). Furthermore, physiologically-relevant neural networks can be constructed *in vitro* using narrowing microchannels (“axon diodes”) that permit unidirectional crossing (Peyrin et al., 2011). Axons growing along the wall of the receiving chamber did not enter the diodes, suggesting that “axonal stiffness” is a limiting factor in turning behavior. Indeed, axon turning frequency decreases with increasing turning angles (Francisco et al., 2007). Accordingly, axons accelerate in narrow channels (funneling effect) where only U-turn is possible: axons grew 20× faster within silicon nitride nanomembrane tubes, 2.7–4.4 μm in diameter (Figures 1D,D′; Froeter et al., 2014).

## Applying Forces to Growth Cones

Unlike passively modulating growth cone behavior through changing its microenvironment, external force application actively stimulates mechanosensitive pathways. Collagen-coated glass microelectrodes were the first tools to pull axons, where chick sensory neurites were towed at 40 μm/h and reached up to 1 mm length (Bray, 1984). Subsequent studies showed that the towing rate was proportional to tension (~1 μm/h per 10 pN) above a force threshold, which depended on the neuron type and the target CAM (Chada et al., 1997). Interestingly, over long timescales, central and peripheral neurons exhibited drastically different (fluid-like vs. solid-like, respectively) responses.

**Figure 2:**
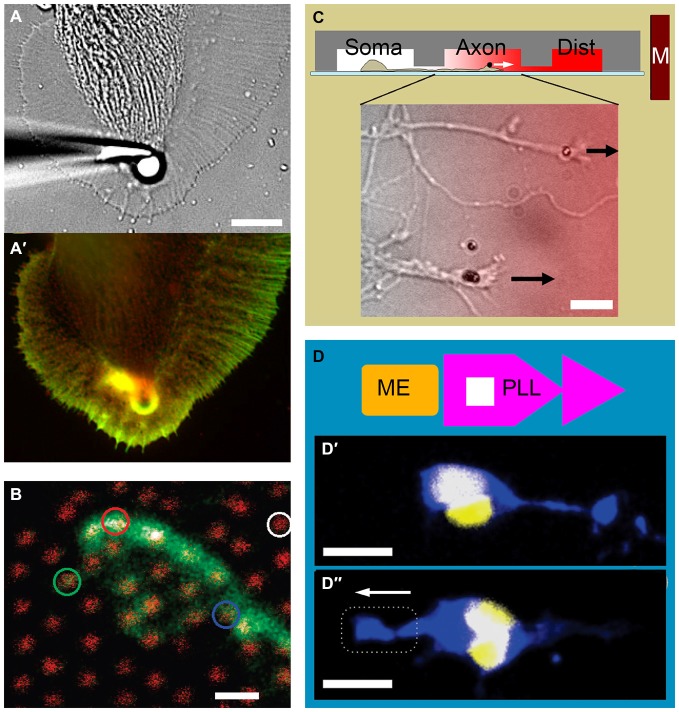
**Examples of growth cone force application and measurement. (A)** Force application via a 5 μm polystyrene bead coated with apCAM, restrained using a glass pipette. **(A′)** Actin (green) and cortactin (red) distribution in an *Aplysia* growth cone is visualized after 7 min of force application. Note the cytoskeletal alignment and the actin-rich arc around the bead. Adapted with permission from Decourt et al. (2009). **(B)** The growth cone of a mouse dorsal root ganglion neuron expressing green fluorescent protein cultured on an array of 40 nm diameter, 4 μm high gallium phosphide nanowires with 1 μm spacing (red). Adapted with permission from Hällström et al. (2010). Copyright 2010 American Chemical Society. **(C)** Three-compartmental microfluidic device for isolating axons and exposing them to linear concentration gradients. Soma, somatic; Axon, axonal; Dist, distal. Growth cones of mouse cortical neurons targeted with neural cell adhesion molecule (NCAM)-functionalized 1.4 μm superparamagnetic beads. Arrows indicate force direction. Overlay color indicates Semaphorin 3A gradient. Adapted from Kilinc et al. (2014). **(D)** Combination of adhesive patterning and force application. The substrate consists of an array of “modified fast-forward” poly-l-lysine patterns (PLL; purple) and embedded ferromagnetic elements (ME; gold). **(D′)** Rat cortical neurons with internalized magnetic nanoparticles polarize in the forward direction in the absence of force. **(D″)** Neuronal polarization is reverted in the presence of magnetic force. Neurons are stained against Tau protein (blue) and 4′,6-diamidino-2-phenylindole (DAPI; yellow). Broken line indicates magnet position. Arrow indicates force direction. Adapted with permission from Kunze et al. (2015). Copyright 2015 American Chemical Society. Scale bars = 10 μm **(A)**, 1 μm **(B)**, 20 μm **(C)**, 16 μm **(D)**.

The last decade witnessed the development of microtechnologies for growth cone force application. Force can be applied indirectly through simply counteracting the force exerted by the growth cone cytoskeleton. This involves targeting a CAM with a microbead that is either restrained with a microneedle (Figures 2A,A′; Decourt et al., 2009), or trapped in a laser beam (Bard et al., 2008). Here, the targeted CAM is coupled to the actin retrograde flow, the constant inward movement of F-actin as it polymerizes at the leading edge and depolymerizes at the inner regions of the growth cone. Restraining the bead interrupts the retrograde flow and diverts the growth cone through locally reorganizing its actin cytoskeleton, followed by the advance of microtubules towards the bead (Schaefer et al., 2008). A bead restrained with a microneedle can exert practically unlimited (yet typically unknown) amounts of force, whereas laser traps operate in a narrow force range (<10 pN). One promising alternative is magnetic tweezers (MTW), where time-modulated forces >1 nN can be applied directly (Kilinc and Lee, 2014).

Classical MTW involves plasma membrane-bound targets. 450 pN acting on integrins (*via* Ø4.5 μm bead) was sufficient to initiate and tow chick forebrain axons (Fass and Odde, 2003), whereas 10–100 pN (Ø40 nm beads) stretched individual filopodia but failed to promote growth cone advance (Pita-Thomas et al., 2015). In contrast, ~10 pN (Ø1.4 μm beads) acting on neural CAM was sufficient to tow mechanically-compromised axons (Kilinc et al., 2014). Recently, researchers started using internalized nanoparticles as MTW handles: upon exposure to magnetic field, nerve growth factor (NGF)-functionalized nanoparticles accumulated in the growing tips of PC12 neurites and affected their orientation (Riggio et al., 2014). Nanoparticles have also been functionalized with agonists of signaling pathways to control axon growth. Localizing Tropomyosin receptor kinase B (TrkB) pathway agonists to growth cone periphery *via* MTW forces interfered with the local F-actin remodeling and affected growth cone advance (Steketee et al., 2011). Finally, axons with internalized nanoparticles can be stimulated in parallel, using micromagnet arrays embedded in the substrate (Kunze et al., 2015). In summary, bead-based microtechnologies, MTW in particular, now offer a wide range of forces to be exerted on growth cones, externally or from within. MTW is becoming more and more accessible to neurobiologists, thanks to the recent progress in particle technology and the emergence of patterned micromagnets.

## Measuring Forces in Growth Cones

A complete understanding of growth cone mechanics requires the characterization of mechanical properties of growth cones and the forces they generate. The stiffness of live *Aplysia* growth cones was mapped out using the atomic force microscope, where the thicker central domain was determined to be 3× and 5× softer than lamellipodia and filopodia, respectively (Xiong et al., 2009). Due to their smaller size, the (visco)elastic properties of mammalian growth cones cannot be characterized with this technique. Instead, local stresses can be estimated from the actin retrograde flow while the growth cone is being compressed (Betz et al., 2011). Once the local stress distribution is known, the internal force field can be calculated. We mentioned that force can be exerted by restraining a microbead bound to a CAM that is coupled to the underlying cytoskeleton. If the bead is restrained using a calibrated optical trap, the exerted force can be determined (Cojoc et al., 2007). Although this measurement can be made at multiple spots by employing optical holograms (Mejean et al., 2009), optical tweezers cannot map out growth cone forces. It is, however, possible to map out traction forces, which the growth cones exert to the substrate *via* focal adhesions. Through time-lapse imaging of fluorescent markers embedded in a soft gel, the strain field (thus the force field) can be measured (Koch et al., 2012; Toriyama et al., 2013; Hyland et al., 2014). Alternatively, neurons can be cultured on micropillar arrays. Force acting on each micropillar can be calculated from its bending, which is a function of its stiffness, height and diameter. Despite being available for over a decade (Tan et al., 2003), this technology could not be applied in neurobiology, due to the high spacing between polymeric pillars. This problem has recently been overcome using arrays of gallium phosphide nanowires (Ø40 nm; 1 μm spacing), which permit force detection at 15 pN resolution (Figure 2B; Hällström et al., 2010).

## Combining Mechanical and Biochemical Stimuli

As growth cones harness mechanical and biochemical stimuli alike, experimental models that separately control the two types of stimuli are sought-after. “Designer” growth cone environments can be created by combining chemical gradients, topographic features, adhesive patterns, and stiffness modulation. For example, growth behavior has been investigated in 2D by presenting neurons with competing topographical (parallel grooves) and chemotactic (immobilized NGF) cues, where the former dominated axon polarization (Gomez et al., 2007a). Interestingly, topographical cues exhibited a strong effect on axon initiation, but a weaker effect on axon elongation after initiation (Gomez et al., 2007b). 3D culture inherently combines the effects of matrix stiffness and nanotopography. As mentioned earlier, self-assembling peptide nanofiber gels now provide independent control on the matrix stiffness (Sur et al., 2013). Effects of topography (maybe also matrix stiffness) and of haptotactic cues can also be compared in 3D by culturing cells on an electrospun nanofiber gel with a gradient of surface-bound laminin (Zander and Beebe, 2014). When the gradient was parallel to the nanofibers, PC12 neurites grew preferentially up the laminin gradient; however, when the gradient was imposed in the perpendicular direction, they were unaffected. These examples show that combinatorial approaches can be very effective in designing growth cone microenvironments.

Direct force application has recently been combined with topographical, chemotactic, and adhesive cues to study neuron polarization and axon growth. Neural stem cells have been cultured on an elastic substrate with microgrooves to compare stretch-induced and microtopography-induced axon orientation and growth (Chang et al., 2013). Neurites stretched parallel to the microchannel geometry grew longer and displayed higher neuronal gene expression, compared to those stretched in the perpendicular direction. We have recently presented a microfluidic device that not only (fluidically) isolates axons from somata, but also exposes them to chemotactic gradients parallel to their elongation direction (Kilinc et al., 2014). Exclusive access to the “axon chamber” facilitated the targeting of growth cones with magnetic beads, leading to parallel MTW force application (Figure 2C). By subjecting axons (and not somata) to a panel of inhibitory molecules, we demonstrated that low pN forces can steer CNS axons towards potent axon repellents, when their molecular motors are locally inhibited. Finally, the competition between force application and substrate patterning has been studied using a microfabricated substrate containing arrays of “fast-forward” adhesive patterns and embedded magnetic elements (Kunze et al., 2015). Low pN forces delivered *via* internalized magnetic nanoparticles reverted the neuron polarization induced by the micropattern (Figures 2D,D′,D″).

## New Tools and Future Outlook

A number of techniques for measuring and applying forces have emerged that can be implemented to study the mechanics of axon growth in the coming years. For example, the stress inside living cells or at the cell-substrate interface can be measured using Förster resonance energy transfer (FRET)-based molecular tension sensors. These sensors have already been incorporated into several focal adhesion proteins, including integrin (Morimatsu et al., 2013), vinculin (Grashoff et al., 2010), and spectrin (Meng and Sachs, 2012), and can report the stress distribution within growth cones in real-time. In addition, nanoparticle tension probes, based on distance-dependent fluorescent quenching of substrate-bound gold nanoparticles, can report tension across individual integrins with submicron resolution (Liu et al., 2014). An emerging technology for massively parallel force application is acoustic tweezers, the manipulation of microparticles using standing acoustic waves (Ding et al., 2012). Similarly, standing magnetic field waves can be generated using a micromagnet array to control the motion of individual magnetic beads in parallel (Li et al., 2013).

Our understanding of the mechanics of axon guidance is in its infancy compared to its biochemistry. Recent and emerging microtechnologies now provide us with a range of tools that can be integrated into sophisticated experimental platforms for closing this gap. The ideal experimental platform will allow us to manipulate and precisely measure the mechanical interactions in and around a growth cone, in a tightly controlled microenvironment. Such a high level of control will help us dissect the mechanotransduction mechanisms in growth cones downstream of other, *e.g*., biochemical, stimuli, in order to reach a more complete understanding of the growth cone behavior.

## Conflict of Interest Statement

The authors declare that the research was conducted in the absence of any commercial or financial relationships that could be construed as a potential conflict of interest.
